# Diagnostic Potential of Evaluation of SDF-1*α* and sRAGE Levels in Threatened Premature Labor

**DOI:** 10.1155/2016/2719460

**Published:** 2016-07-31

**Authors:** Rafał Rzepka, Barbara Dołęgowska, Aleksandra Rajewska, Daria Sałata, Marta Budkowska, Sebastian Kwiatkowski, Andrzej Torbé

**Affiliations:** ^1^Department of Obstetrics and Gynecology, Pomeranian Medical University, Ulica Powstańców Wielkopolskich 72, 70-111 Szczecin, Poland; ^2^Department of Laboratory Diagnostics and Molecular Medicine, Pomeranian Medical University, Ulica Powstańców Wielkopolskich 72, 70-111 Szczecin, Poland

## Abstract

Preterm birth remains the most prevalent cause of neonatal morbidity. This study aimed to evaluate the diagnostic value of SDF-1*α*, resistin, secretory RAGE (sRAGE), and endogenous secretory RAGE (esRAGE) in preterm labor. A total of 211 pregnant women participated in the study. Group A contained 72 women between 22 and 36 weeks of gestation, with premature labor, who finally had preterm birth. Group B contained 66 women in labor between 37 and 41 weeks of gestation. Women in group A had lower SDF-1*α* and sRAGE levels than those in group B. Moreover, in group A, SDF-1*α* and sRAGE levels were correlated with the latency period from the occurrence of premature labor symptoms until delivery. Sensitivity and specificity of studied parameters for prediction of preterm birth were 95% and 40% for SDF-1*α* and 51.3% and 93.5% for sRAGE, respectively. The prognostic value of plasma SDF-1*α* and sRAGE levels was comparable with that of cervical length ultrasound measurement and serum C-reactive protein levels. We conclude that SDF-1*α* and sRAGE appear to play a major role in the diagnosis of preterm birth and its evaluation could be convenient and useful for predicting preterm birth.

## 1. Introduction 

In developed countries, premature birth is the leading cause of neonatal mortality and morbidity, with disability as a consequence [1]. An extremely high neonatal risk is associated with delivery between 22 and 26 weeks of gestation. A total of 65% of neonates born in such early pregnancies die within 30 days as patients of intensive neonatal care wards [2].

Preterm delivery can be iatrogenic or spontaneous. Iatrogenic preterm birth is the result of medical intervention, usually due to fetal and/or maternal conditions (e.g., fetal growth restriction, and preeclampsia). Spontaneous preterm labor and delivery are a heterogeneous condition with many triggers and risk factors, including maternal genital tract hemorrhage, cervical dysfunction, idiopathic uterine contractions, malnutrition, multifetal pregnancy, and spontaneous rupture of the fetal membranes [3, 4].

The most prevalent cause of spontaneous preterm labor is local or generalized infection [5–7]. Infection leads to activation of the immune system via toll-like receptors and cytokine overproduction. This leads to an increase in the activity of prostaglandins and metalloproteinases, which are cardinal initiators of preterm uterine activity and/or preterm premature rupture of the membranes [8–10]. Susceptibility to intrauterine infection is inversely proportional to gestational age [11]. In cases of delivery between 22 and 24 weeks of gestation, the risk of chorioamnionitis is as high as 94.4% and decreases to only 3.8% at term [12]. Interestingly, the same vaginal bacteria can initiate preterm labor at 24 weeks of gestation, while at the 36th week these bacteria are not able to cause any disease [13]. The cause of this phenomenon still remains unclear.

Stromal cell-derived factor 1*α* (SDF-1*α*) is a chemokine from the CXC group. SDF-1*α* is produced mostly in bone marrow stromal cells and epithelial cells of the pancreas, spleen, ovary, small intestine, and other organs [14]. Expression of SDF-1*α* is also found in human trophoblasts [15]. SDF-1*α* probably facilitates trophoblast invasion and spiral artery remodeling. SDF-1*α* enhances vascular endothelial growth factor expression in pregnancy and also participates in neovascularization. CXC chemokine receptor type 4 (CXCR4) activation by SDF-1*α* is one among many aspects of induction of maternal-fetal immune tolerance to allow proper development of pregnancy [15, 16].

Secretory receptors for advanced glycation end products (sRAGE) and endogenous secretory receptors for advanced glycation end products (esRAGE) belong to the group of negative forms of RAGE [17]. Ligand-RAGE interaction increases oxidative stress and stimulates production of nuclear kappa-B factor (NF-*κ*B). NF-*κ*B activates the expression of genes for cytokines, such as tumor necrosis factor- (TNF-) *α*, interleukin- (IL-) 1 and IL-6, and genes for the adhesive proteins VCAM-1 and ICAM-1, which participate in the inflammatory response [18]. After its loss of affinity for heparan sulfate, the sRAGE-ligand complex is released to circulating blood. Finally, the sRAGE-ligand complex becomes degraded in the spleen or liver. In blood vessels, sRAGE and esRAGE play an important protective role against toxic effects of ligand-RAGE complexes, reducing the inflammatory response [19, 20].

Resistin (RE) is an adipokine from the family of cysteine-rich proteins, referred to as resistin-like molecules. In humans, resistin is mainly produced in inflammatory cells of peripheral blood, such as monocytes and macrophages [21]. Resistin gene expression can be modulated via some inflammatory signal molecules, with nuclear kappa-B factor and cytokines among them [22]. IL-6, TNF-*α*, and lipopolysaccharide stimulate the expression of the resistin gene [23]. RE plays a role in some inflammatory diseases. An increased level of RE was found not only in synovial fluid in patients suffering from rheumatoid arthritis, but also in blood plasma of patients with nonspecific inflammatory diseases [24, 25]. Some researchers have postulated the importance of resistin in premature labor [26].

Nevertheless, the role of the above-mentioned biochemical factors in premature labor is still unclear. Changes in their concentrations probably affect the inflammatory response in the course of premature labor. Such fluctuations can also moderate susceptibility of the maternal-fetal unit to infection.

## 2. Objectives

The objectives of this research were to compare plasma SDF-1*α*, RE, sRAGE, and esRAGE concentrations between women who had premature birth and those at term. Additionally, we aimed to assess the prognostic value of plasma SDF-1*α*, RE, sRAGE, and esRAGE concentrations for preterm birth in women presenting with symptoms of premature labor. Finally, this study aimed to compare the usefulness of determining SDF-1*α*, RE, sRAGE, and esRAGE levels with cervical length by ultrasound and serum C-reactive protein level measurement in clinical practice.

## 3. Materials and Methods

The study was conducted in the Department of Obstetrics and Gynecology and in the Department of Laboratory Diagnostics and Molecular Medicine of the Pomeranian Medical University in Szczecin, Poland, from January 01, 2013, to December 30, 2015. A total of 211 pregnant women were included in the study and then assigned to two study groups and two control groups. Study group A contained 72 women with a gestational age between 22 and 36 weeks, presenting with symptoms of premature labor, who finally delivered prematurely. Study group B consisted of 66 women in labor between 37 and 41 weeks of gestation. Control group C contained 40 women at a gestational age from 22 to 37 weeks without signs or symptoms of threatened premature labor. Control group D comprised 33 women at a gestational age from 37 to 41 weeks with no symptoms of onset of spontaneous labor.

Criteria for inclusion in group A were as follows: (i) spontaneous uterine activity, which was visible in cardiotocography, lasting for at least 2 hours, with at least four uterine contractions per hour, with concomitant cervical effacement to less than 25 mm in ultrasound measurement, between 22 and 36 weeks of gestation; (ii) preterm premature rupture of the membranes between 22 and 36 weeks of gestation, confirmed with a positive result of a test for the presence of insulin-like growth factor binding protein-1 in cervicovaginal discharge; and (iii) completion of delivery before 36 weeks of gestation. The criterion for inclusion in group B was spontaneous uterine activity as shown by cardiotocography after 37 weeks of gestation, lasting for at least 2 hours, with at least four uterine contractions per hour with concomitant cervical effacement to less than 25 mm in ultrasound measurement. Criteria for inclusion in group C were as follows: (i) gestational age between 22 and 36 weeks, (ii) absence of spontaneous uterine activity as shown by cardiotocography, and (iii) cervical length longer than 25 mm in ultrasound measurement. The criterion for inclusion in group D was the absence of uterine activity as shown by cardiotocography in pregnancy lasting more than 37 weeks.

Patients with multiple pregnancies, fetal malformations, preeclampsia, and systemic diseases were excluded from the study. The detailed characteristics of the study groups are shown in Table 1.

Not later than 2 hours after admission to the department, peripheral maternal blood was sampled from the ulnar vein and placed into tubes containing EDTA-K2. After centrifugation (10 minutes at 5000 ×g), plasma samples were stored at −80°C until analyses of SDF-1*α*, RE, sRAGE, and esRAGE concentrations could be performed. Immunoassay methods were used to measure SDF-1*α*, sRAGE, esRAGE, and resistin concentrations. The human CXCL12/SDF-1 alpha ELISA method (R & D Systems) was used for quantitative measurement of human SDF-1*α*, with a calibration range of 156–10,000 pg/mL, and a limit of detection of 47 pg/mL. Human resistin ELISA (Bio Vendor Research and Diagnostic Products) was used for quantitative measurement of human resistin levels, with a calibration range of 1000–50,000 pg/mL and a limit of detection of 12 pg/mL. Human sRAGE ELISA (Bio Vendor Research and Diagnostic Products) was used for quantitative measurement of human sRAGE with a calibration range of 50–3200 pg/mL, and a limit of detection of 19.2 pg/mL. Human esRAGE ELISA (Cusabio, CSB-E15773 h) was used for quantitative measurement of human esRAGE. The calibration range for esRAGE was 0.625–40 ng/mL, with a limit of detection of 0.156 ng/mL. Coefficients of variation for the assays are shown in Table 2.

All women included in the study groups also had their white blood cell count and C-reactive protein (CRP) levels measured. In groups A and C, the ultrasound cervical length was assessed with a vaginal probe, which was placed in the vestibule of the vagina. The arithmetic mean of three subsequent measurements was used in the study. In group A, intravenous inflow of fenoterol at a dosage ranging from 0.0035 to 0.005 mg/min was administered as a tocolytic agent, until inhibition of uterine contractions. The pregnant women were also administered betamethasone in two 12 mg doses with 24-hour intervals to accelerate fetal lung maturation. Group A was also categorized into subgroups by the duration of pregnancy from the diagnosis of premature labor up to delivery, with a 48-hour cut-off point.

The study was approved by the Bioethical Committee of the Pomeranian Medical University (KB-0012/121/12). All women gave their written informed consent prior to their inclusion in the study.

### 3.1. Statistical Analysis 

Statistical evaluation was performed using Statistica 12.5 PL software for Windows. The distribution of variables was checked using the nonparametric Shapiro-Wilk* W* test, and, according to its results, values were further analyzed. A level of significance (*p*) lower than 0.05 was considered significant. For presentation of nonnormally distributed variables, the number of patients (*N*), range in values (minimum to maximum), median (Me), and first and third quartile values (Q1–Q3) were included in descriptive statistics. To assess the differences between analyzed parameters between two groups, the Mann-Whitney test for unpaired variables was used. For statistical analysis of the relationship between X and Y, correlation coefficients were estimated using Spearman's test. Receiver operating characteristic (ROC) curve analyses were used to determine the cut-off point, as well as the predictive value of tests, and their sensitivity, specificity, and positive and negative predictive values. The accuracy was also determined. Comparison of the area under the curve was used for comparison of diagnostic tests.

## 4. Results

Most parameters in our study had a nonnormal distribution (Shapiro-Wilk test, *p* < 0.05). To exclude the potential effect of gestational age on plasma SDF-1*α*, resistin, sRAGE, and esRAGE concentrations, we compared their levels between the control groups. There was no significant difference in plasma SDF-1*α*, resistin, sRAGE, and esRAGE concentrations between the control groups (Table 3).

**Figure 1:**
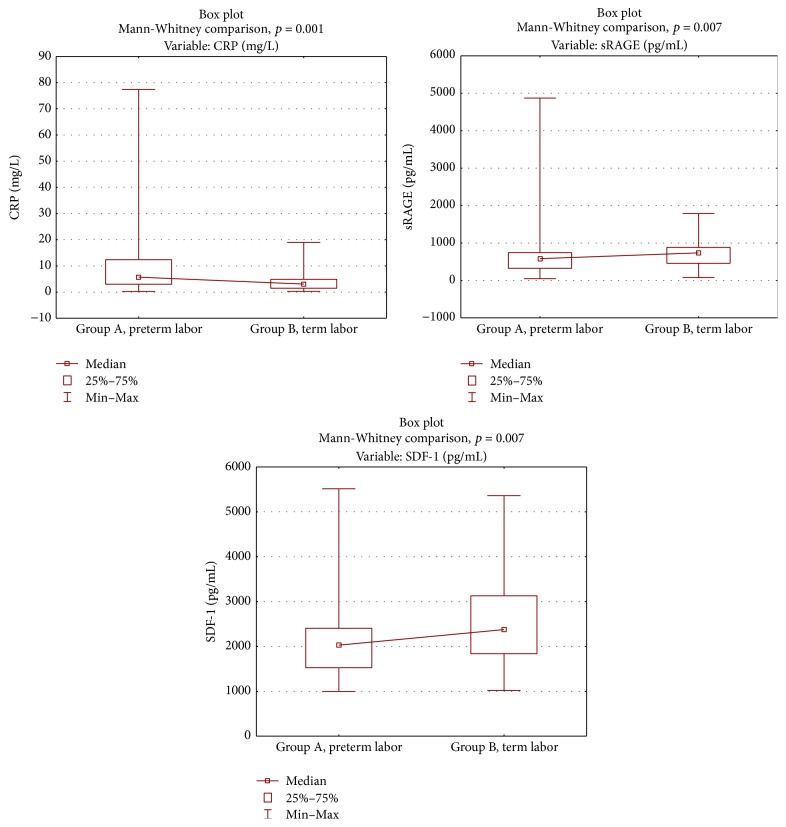
Comparison of SDF-1*α*, CRP, and sRAGE between the groups. The Mann-Whitney* U* test was used for comparison between groups.

**Figure 2:**
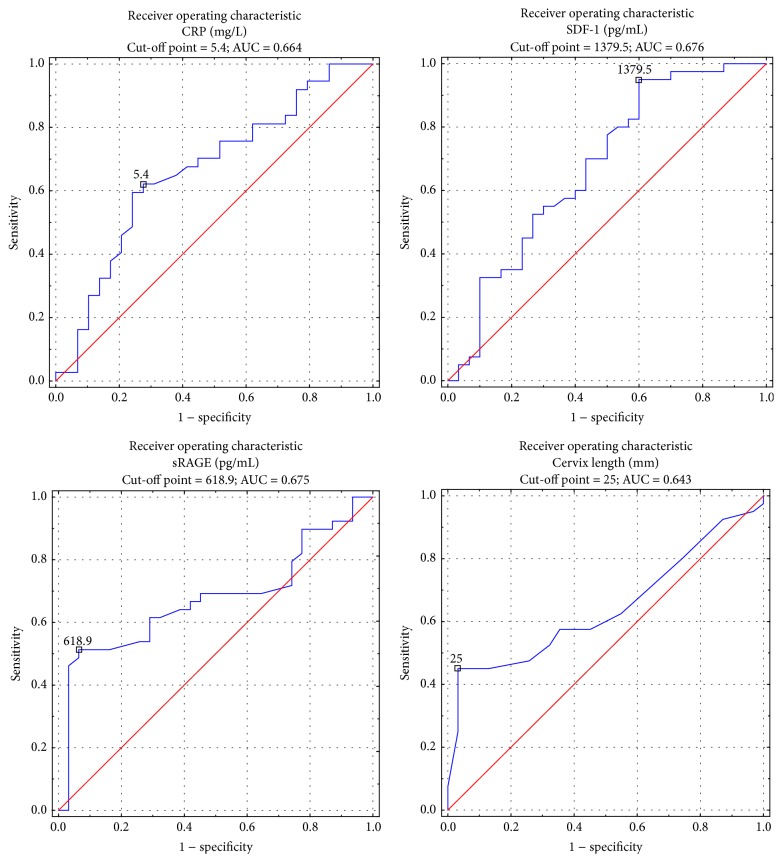
ROC curves analysis of CRP, SDF-1*α*, sRAGE, and cervix length for time from symptoms until delivery.

**Figure 3:**
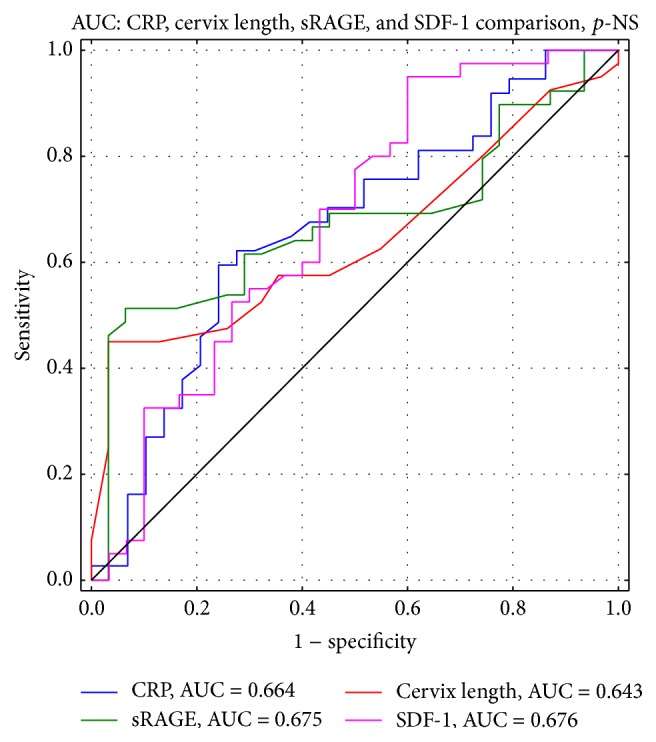
Group A. Areas under the ROC curves comparison for CRP, cervix length, sRAGE, and SDF-1*α*.

Data on the descriptive statistics in the study groups is shown in Table 4. Plasma RE and esRAGE levels and the WBC were not significantly different between the study groups (Table 5). Plasma SDF-1*α* and sRAGE concentrations were significantly lower in group A than in group B (Figure 1). In group A, there were positive correlations of the latency period from the onset of premature labor symptoms until delivery with plasma sRAGE and SDF-1*α* concentrations and cervical length measured in vaginal ultrasound (*r* = 0.301, *p* = 0.01; *r* = 0.301, *p* = 0.01; *r* = 0.247, *p* = 0.04, resp.). A negative correlation between the duration of the latency period and plasma CRP concentrations was also found. Women in group A who delivered in less than 48 hours from the onset of premature labor had lower plasma SDF-1*α* and sRAGE levels than those with a longer latency period (Me = 1765 pg/mL versus 2720 pg/mL; Me = 366.3 pg/mL versus 636.9 pg/mL, resp.). Comparison of values of parameters that depended on the duration of the latency period is shown in Table 6. ROC curve analysis showed that plasma SDF-1*α* concentrations higher than 1379.5 pg/mL and sRAGE higher than 618.9 pg/mL indicated a low risk of delivery in less than 48 hours from the onset of symptoms. The sensitivity of SDF-1*α* was as high as 95%, but its specificity reached only 40%, similar to sRAGE, with a sensitivity of 93.5% and specificity of 51.3%. Ultrasound cervical length measurement, with a cut-off point 25 mm, had a sensitivity of 45% and specificity of 96.8%. Sensitivity of plasma CRP concentrations was 62.2% and specificity reached 72.4%. ROC analysis is shown in Figure 2. Comparison of the area under the ROC curve among cervical length, and plasma CRP, SDF-1*α*, and sRAGE levels did not show significant differences. Prognostic values of SDF-1*α* and sRAGE tests were comparable with those of ultrasound calculation of cervical length and CRP levels (Figure 3).

## 5. Discussion 

This study showed that plasma levels of SDF-1*α*, sRAGE, esRAGE, and resistin were independent of gestational age, which enabled analysis of their changes in groups A and B as markers of premature labor.

In our study, plasma SDF-1*α* concentrations were lower in women who gave birth prematurely. Modulation of immune system function is necessary for normal development of pregnancy [27–29]. The maternal-fetal unit needs to increase its production of Th2-dependent anti-inflammatory cytokines, such as IL-4 and IL-10, and to reduce production of Th1-dependent proinflammatory cytokines, including interferon-gamma and TNF-*α* [27–29]. Piao et al. showed that the axis SDF1/CXCR4 participates in Th1/Th2-dependent cytokine production, especially enhancing synthesis of those with anti-inflammatory function. Inhibition of SDF-1*α* receptor changes the balance towards overproduction of Th1-dependent proinflammatory cytokines [30]. Other researchers have also suggested that abnormalities of immunological interactions between pregnant woman and the trophoblast can result in miscarriage, preterm delivery, or intrauterine growth restriction [31–35]. Some reports have shown SDF1-*α* deficiency as a source of complications in pregnancy, especially preeclampsia. Song et al. found decreased CXCL12 gene expression in women with severe preeclampsia in preterm pregnancy compared with healthy pregnant women [36].* In vitro* experiments in sheep showed that stimulation of trophoblast cells with CXCL12 increased the expression of mRNA for vascular endothelial growth factor and fibroblast growth factor 2, which are important for placentation [37]. Low expression of SDF-1*α*/CXCR4 in placentas of women with preeclampsia appears to confirm the role of CXCL12/CXCR4 in this type of complication in pregnancy [38]. In contrast, Laudanski et al. analyzed plasma SDF-1*α* levels in 109 women with threatened premature labor and delivery and did not find any significant difference compared with women who delivered at term [34]. Similarly, another study showed no difference in plasma SDF-1*α* concentrations between women who delivered prematurely and those who delivered at term [35]. However, increased SDF-1*α* plasma levels have been shown to be present in premature neonates [35]. Tseng et al. showed the protective function of increased SDF-1*α* concentrations in amniotic fluid in the second trimester of pregnancy. They showed a significantly lower prevalence of preterm birth in women who had higher amniotic fluid SDF-1*α* levels [16].

Our results appear to be consistent with molecular theories of the SDF-1/CXCR4 axis. Changes in homeostasis towards overproduction of Th1-dependent cytokines as an effect of SDF-1*α* deficiency can activate the proinflammatory cascade, resulting in preterm delivery. The finding of a correlation between plasma SDF-1*α* levels and the duration of the latency period from the onset of premature labor to completed delivery suggests a protective role of this factor.

The role of negative forms of RAGE receptors is currently the focus of research. Studies have mostly confirmed a protective function of sRAGE against inflammatory diseases [39–45]. In our study, there were lower plasma sRAGE levels in group A, containing patients who gave birth prematurely. The ligand-RAGE receptor interaction enhances oxidative stress via NADPH oxidase activation and some transcription activating factors. These factors are mainly NF-*κ*B and mitogen-activated protein kinase [18, 46]. After its release, active NF-*κ*B moves into the cell nucleus to activate expression of genes for proinflammatory cytokines, such as TNF-*α*, Il-1, and Il-6, and for the adhesive proteins VCAM-1 and ICAM-1, of which their products contribute to the inflammatory response [46, 47]. Inhibition of this process by negative isoforms of receptors reduces the intensity of inflammation, while deficiency of negative RAGE forms results in excessive activation of the inflammatory response.

The protective role of increased sRAGE levels against preterm delivery was also suggested by Bastek et al. in their analysis of 529 women with premature labor [48]. They found that lower sRAGE concentrations were related to earlier delivery. They concluded that sRAGE levels could be a useful marker of preterm birth [48]. Similarly, another study showed decreased RAGE receptor concentrations in women with overt chorioamnionitis [49]. Germanová et al. also suggested a protective function of sRAGE after finding decreased plasma sRAGE levels in patients suffering from premature labor and preeclampsia compared with those with an uncomplicated pregnancy [50, 51]. They concluded that further studies are required to demonstrate the usefulness and importance of sRAGE in diagnosis of preterm labor. Consistent with our results, Hájek et al. observed lower sRAGE concentrations in women with premature labor and preterm premature rupture of the membranes than in healthy pregnant women. They postulated that even just the occurrence of preterm labor symptoms is associated with reduced levels of sRAGE and that sRAGE can be a new marker for prediction of preterm delivery [52]. In our study, we found lower sRAGE concentrations in women who delivered prematurely. We observed a positive correlation between plasma sRAGE levels and pregnancy duration. This finding strongly suggests that reduced sRAGE levels enhance the inflammatory response in women with premature labor. Research from other branches of medicine has suggested that low sRAGE concentrations augment inflammation, indicating that sRAGE is an independent risk factor of approaching intensification of the disease [39, 53–55]. We previously found that decreased sRAGE levels were an independent predictive parameter of preterm delivery in women with threatened premature labor with intact membranes [56, 57].

Originally, resistin was only believed to play a part in some metabolic processes. Currently, there is a lot of evidence that resistin is associated with the inflammatory response [58–60]. Some researchers suggest that, in uncomplicated pregnancies, resistin levels steadily increase. Higher resistin concentrations are usually found at term [61]. However, in our study, we did not find higher resistin levels in patients at term. Some researchers have observed increased plasma RE levels in premature labor with accompanying chorioamnionitis [26]. However, recently, Kominiarek et al. analyzed plasma adiponectin, resistin, and leptin concentrations and did not find any significant differences in these parameters between women with premature labor and those who actually give birth prematurely [62] as in our study groups. We also did not find a correlation between resistin concentrations and the latency period from the onset of symptoms of threatened premature labor until the actual beginning of labor. Our study indicated the inadequacy of resistin concentrations as a diagnostic parameter in premature labor.

The prevalence of preterm birth in Europe and in the United States has remained constant for many years [63]. However, there is still no ideal marker for prediction of preterm delivery. Ultrasound measurement of cervical length, plasma CRP levels, and fibronectin concentrations in cervicovaginal discharge are currently the most commonly used markers in such circumstances [64–68]. In our study, we found associations between plasma CRP, sRAGE, and SDF-1*α* levels and cervical length and pregnancy duration. Therefore, we decided to calculate the predictive value of each marker for completion of delivery in 48 hours from the onset of threatened premature labor symptoms. For practical reasons, a 48-hour period was set, as necessary for effective use of prenatal corticosteroid therapy. ROC curve analysis showed that the values of sensitivity and specificity of SDF-1*α* and sRAGE plasma concentrations were similar with those of plasma CRP concentrations and cervical length ultrasound measurements. Analysis and comparison of the areas under the ROC curves did not show superiority of any of the common premature labor markers over the new markers that we investigated. Because of major limitations in our study group size, alternative use of plasma SDF-1*α* and sRAGE level assessment instead of plasma CRP levels or cervical length measurement is not yet unquestionable. Nevertheless, use of other premature labor markers, such as damage-associated molecular patterns and the RAGE receptors, appears reasonable [69]. Analysis of the chemokines in the context of pathogenesis of premature labor, with special attention to those modulating Th1- and Th2-dependent immune responses, could be a promising research option.

## 6. Conclusions 

Decreased plasma SDF-1*α* and sRAGE concentrations in women who deliver prematurely suggest the importance of deficiency of these factors for preterm labor. Further investigations on this issue are required. Additionally, significantly decreased plasma SDF-1*α* and sRAGE levels in women who deliver in less than 48 hours from the onset of threatened premature labor suggest that assessment of these markers is useful for diagnosing this complication.

Analysis and comparison of the areas under the ROC curves for cervical length and plasma CRP, SDF-1*α*, and sRAGE levels in group A showed that none of the investigated parameters is an ideal marker of preterm delivery. However, the clinical value of assessment of SDF-1*α* and sRAGE concentrations is comparable with that of cervical length and plasma CRP levels. Further research is required in the field of chemokines and negative RAGE receptor isoforms in the diagnosis of premature labor.

## Figures and Tables

**Table 1:** General characteristic of study groups.

Parameter	Group A preterm labor	Group B term labor	*p* value
Number of women	72	66	—
Age (years)	30.17 ± 6.22	27.86 ± 5.94	0.04
Gestational age (weeks)	31.72 ± 3.35	38.87 ± 1.09	0.00
Parity	2 ± 1	2 ± 1	NS
Birth weight (g)	1945.45 ± 723.32	3288.09 ± 413.15	0.00
Smoker (*N*)	10	8	NS
Nonsmoker (*N*)	62	58	
Place of residence, city (*N*)	45	40	NS
Place of residence, village (*N*)	27	26	
Excellent socioeconomic status (*N*)	34	30	NS
Mediocre socioeconomic status (*N*)	38	36	

**Table 2:** Coefficients of variation for the ELISA assays.

Assay	Coefficient of variation	
	Intra-assay (%)	Interassay (%)
SDF-1*α*	3.4	9.4
RE	5.2	7.0
sRAGE	4.00	7.15
esRAGE	5.20	8.50

SDF-1*α*: stromal cell-derived factor 1*α*; RE: resistin; sRAGE: secretory receptors for advanced glycation end products; esRAGE: endogenous secretory receptors for advanced glycation end products.

**Table 3:** Mann-Whitney comparison of study parameters between control groups.

Parameter	Control group C, preterm pregnancy					Control group D, term pregnancy					*p*
	*N*	Min–max	Q1	Q3	Median	*N*	Min–max	Q1	Q3	Median	
SDF-1*α* (pg/mL)	40	1018–3437	1547	2654	2182	33	1354–5362	1855.5	3477.5	2678	NS
RE (pg/mL)	40	4075–17750	6189	12120	8768	33	3292–121500	7061	14733	1019	NS
sRAGE (pg/mL)	40	128.7–1216	551.7	870	765.1	33	85.64–1947	285.18	887.55	714.4	NS
esRAGE (pg/mL)	40	345.0–940.0	432.1	582.8	499.3	33	400.0–1040	488.4	599.6	539.3	NS

SDF-1*α*: stromal cell-derived factor-1*α*; RE: resistin; sRAGE: secretory receptors for advanced glycation end products; esRAGE: endogenous secretory receptors for advanced glycation end products; Q1: quartile 1; Q3: quartile 3; min: minimum; max: maximum.

**Table 4:** Descriptive statistics of the study groups.

Parameter	Group A, preterm labor					Group B, term labor				
	*N*	Min–max	Q1	Q3	Median	*N*	Min–max	Q1	Q3	Median
WBC (10^9^/L)	72	3.32–25.4	9.96	14.4	12.0	65	7.14–21.08	9.97	14.0	11.9
CRP (mg/L)	72	0.3–19.0	1.5	4.9	3.0	65	0.2–77.3	2.7	11.8	5.8
SDF-1*α* (pg/mL)	72	1000–5515	1553	2418	2031	65	1018–5362	1836	3128	2374
RE (pg/mL)	72	3958–52950	6787	11180	8214	65	3277–131800	7022	14640	9739
sRAGE (pg/mL)	72	48.9–4872	333.2	683.4	456.0	65	77.4–1787.7	458.4	882.9	736.9
esRAGE (pg/mL)	72	230.0–958.8	463.05	604.7	526.6	65	345.0–1096.8	472.1	599.2	532.1

WBC: white blood cells; CRP: C-reactive protein; SDF-1*α*: stromal cell-derived factor-1*α*; RE: resistin; sRAGE: secretory receptors for advanced glycation end products; esRAGE: endogenous secretory receptors for advanced glycation end products; Q1: quartile 1; Q3: quartile 3; min: minimum; max: maximum.

**Table 5:** Comparison of study parameters between study groups.

Parameter	Rank-sum group A, preterm labor	Rank-sum group B, term labor	*U*	*Z*	*p*
WBC (10^9^/L)	3454	3101.0	1561.5	−0.346	NS
CRP (mg/L)	3683	1168	640.00	−3.148	0.001
SDF-1*α* (pg/mL)	3675	3346	1190	2.682	0.007
RE (pg/mL)	3509	3872	1493	1.730	NS
sRAGE (pg/mL)	4068	4709	1583.5	2.671	0.007
esRAGE (pg/mL)	3755.5	3625.5	1739.5	0.451	NS

WBC: white blood cells; CRP: C-reactive protein; SDF-1*α*: stromal cell-derived factor-1*α*; RE: resistin; sRAGE: secretory receptors for advanced glycation end products; esRAGE: endogenous secretory receptors for advanced glycation end products; *U*: Mann-Whitney *U* test; *Z*: Mann-Whitney *Z* test; *p*: Mann-Whitney level of significance.

**Table 6:** Comparison of study parameters between subgroups in the group of preterm labor.

Parameter	Group A1 < 48 h					Group A2 > 48 h					*p*
	*N*	Min–max	Q1	Q3	Median	*N*	Min–max	Q1	Q3	Median	
CL (mm)	31	9–32	11	21	14	41	8–35	12	31	19.5	0.04
WBC (10^9^/L)	31	3.32–25.4	10.19	14.42	13.09	41	6.8–21.9	9.7	14.1	11.1	NS
CRP (mg/L)	31	0.4–77.3	5.3	14.4	7.6	41	0.2–41.7	2.3	8.3	4.45	0.01
SDF-1*α* (pg/mL)	31	1000–5515	1200	2192	1765	41	1101–4282	1766	2720	2168	0.01
RE (pg/mL)	31	4223–30060	6732	11830	8572	41	3958–52950	6843	10530	8035	NS
sRAGE (pg/mL)	31	48.99–4872	324.0	567.0	366.3	41	176.5–1125	333.6	760.1	636.9	0.01
esRAGE (pg/mL)	31	231.9–958.8	475.7	606.5	535.7	41	230.0–915.2	453.9	591.9	522.9	NS

CL: cervix length; WBC: white blood cells; CRP: C-reactive protein; SDF-1*α*: stromal cell-derived factor-1*α*; RE: resistin; sRAGE: secretory receptors for advanced glycation end products; esRAGE: endogenous secretory receptors for advanced glycation end products; Q1: quartile 1; Q3: quartile 3; min: minimum; max: maximum; *p*: Mann-Whitney level of significance.
